# When it’s needed most: a blueprint for resident creative writing workshops during inpatient rotations

**DOI:** 10.1186/s12909-021-02935-x

**Published:** 2021-10-20

**Authors:** Lauren Michelle Edwards, Yeuen Kim, Matthew Stevenson, Tyler Johnson, Nora Sharp, Anna Reisman, Malathi Srinivasan

**Affiliations:** 1 Division of Primary Care and Population Health, Stanford University School of Medicine, 960 North San Antonio Road, Suite 101, Los Altos, CA 94022 USA; 2 Division of Primary Care and Population Health, Stanford University School of Medicine, Palo Alto, CA USA; 3 Division of Primary Care and Population Health, Palo Alto Veterans Administration Hospital, Palo Alto, CA USA; 4 Division of Hematology and Oncology, Stanford University School of Medicine, Palo Alto, CA USA; 5 Stanford Center for Asian Health Research and Education, Stanford University School of Medicine, Palo Alto, CA USA; 6 Computational and Systems Biology Interdepartmental Program, University of California, Westwood, Los Angeles, CA USA; 7 Department of Internal Medicine (General Medicine), Yale School of Medicine, New Haven, CT USA

## Abstract

**Background:**

Narrative Medicine may mitigate physician burnout by increasing empathy and self-compassion, and by encouraging physicians to deeply connect with patient stories/experiences. However, Narrative Medicine has been difficult to implement on hectic inpatient teaching services that are often the most emotionally taxing for residents.

**Objective:**

To evaluate programmatic and learner outcomes of a novel narrative medicine curriculum implementation during inpatient medicine rotations for medical residents. Programmatic outcomes included implementation lessons. Learner outcomes included preliminary understanding of impact on feelings of burnout. Additionally, we developed a generalizable narrative medicine framework for program implementation across institutions.

**Methods:**

We developed and implemented a monthly 45-min Narrative Medicine workshop on Stanford’s busiest and emotionally-demanding inpatient rotation (medical oncology). Using the Physician Wellbeing Inventory (PWBI, range 1–7; 3–4 = high burnout risk; ≥4, high burnout), we anonymously assessed resident burnout during pre-implementation control year (2017–2018, weeks 1 and 4), and implementation year (2018–2019, weeks 1 and 4). We interviewed program directors and facilitators regarding curriculum implementation challenges/facilitators.

**Results:**

Residents highly rated the narrative medicine curriculum, and the residency program renewed the course for 3 additional years. We identified success factors for programmatic success including time neutrality, control of session, learning climate, building trust, staff partnership, and facilitators training. During control year, resident burnout was initially high (*n* = 16; mean PBWI = 3.0, SD: 1.1) and increased by the final week (*n* = 15; PBWI = 3.4, SD: 1.6). During implementation year, resident burnout was initially similar (*n* = 13; PBWI = 3.1, SD: 1.9) but did not rise as much by rotation end (*n* = 24; PBWI = 3.3, SD: 1.6). Implementation was underpowered to detect small effect sizes. Based on our our experience and literature review, we propose an educational competency framework potentially helpful to facilitate inpatient narrative medicine workshops, as a blueprint for other institutions.

**Conclusions:**

Inpatient Narrative Medicine is feasible to implement during a challenging inpatient rotation and may have important short-term effects in mitigating burnout rise, with more study needed. We share teaching tools and propose a competency framework which may be useful to support development of inpatient narrative medicine curricula across institutions.

**Supplementary Information:**

The online version contains supplementary material available at 10.1186/s12909-021-02935-x.

## Introduction

Narrative medicine has been shown to mitigate physician burnout – emotional exhaustion, depersonalization, feelings of reduced accomplishments – by increasing personal resiliency [1, 2] In narrative medicine workshops, participants are exposed to literary techniques, and guided to write about their own and their patient’s experiences in response to themes, using interpretive literary techniques. Narrative Medicine techniques may increase physician empathy and self-compassion [3], encourage physicians to better connect with their patients’ stories, and even act on their behalf [3–5]. Participation in narrative medicine may also improve self-knowledge, peer support, and be perspective-widening – to counter perfectionism, exhaustion, isolation, and depersonalization [6].

Narrative medicine workshops have been difficult to implement on hectic inpatient teaching services, especially during time-intensive and emotionally demanding services, when, arguably, they are most needed [7–9]. At our institution, 40% of internal medicine residents experienced high risk of burnout on their inpatient oncology rotation [10]. Here, residents care for extremely ill patients and their families, who are constantly facing serious medical issues and their own mortality.

In 2018–2019, we implemented and evaluated a narrative medicine workshop during inpatient oncology, and assessed program and learner outcomes. We assessed the short-term effects on resident burnout, in comparison to historical controls. We share lessons learned in developing and implementing this program, identify core and advanced educator competencies, and share teaching tools including examples of systems-based and individual session challenges and approaches.

## Methods

### IRB

This project was considered exempt by the Stanford Institutional Review Board (protocol #41690).

### Funding

This project was supported with funding from the Stanford Teaching and Mentoring Academy (grant #1175550–156-AABKS).

### Med-X rotation

Stanford medicine residents participate in the inpatient oncology rotation (Med-X) during their internship, and once during their 2nd or 3rd year. Med-X (“Med Ten”) is a four-week rotation, with average census of fifteen patients. Teams include an oncology attending, 2 residents, 2 interns and 0–3 medical students. All team members except attendings participated in workshops.

#### Inpatient narrative medicine workshop

For the past 7 years, we have been conducting monthly 1.5 to 2-h narrative medicine workshops at Stanford Hospital and Palo Alto Veterans Administration Hospital during outpatient educational half-days. Residents participate three times per year as interns, and 3–6 times per year as senior residents. Residents read clinically themed prose/poetry, discussed their experiences, responded to a writing prompt for 30 min, then optionally shared their work during discussion. Three faculty alternate as facilitators. One facilitator has post-graduate training in Narrative Medicine and trained the other two faculty facilitators. The outpatient workshop has been renewed yearly, based on high resident ratings. We customized this outpatient workshop for the inpatient setting, with curriculum design following Kern’s Model [11] and key educational concepts from Skeff’s educational framework [12], attending to several developmental principles:

- **Time neutrality:** We worked with program leadership and substituted this workshop for a noon research conference during the last week of the Med-X rotation.
- **Control of session:** For residents to reflect, empathize, share and write, they needed protected time. We partnered with the Med-X charge nurses and unit secretaries to hold all non-essential pages.
- **Learning Climate:** To set an inviting tone, we used a comfortable private space at the cancer center’s outdoor garden proximal to the inpatient floor. Lunch was provided. Facilitators started with several quiet, meditative minutes to help residents transition from patient care. Personal/team respect and privacy were emphasized during each session.
- **Anonymity:** Participants were informed that sharing their writing was voluntary, and that no anecdotes or narratives would be shared outside the group without their permission.
- **Adaptability:** Facilitators were flexible about start times to allow for rounding or urgent clinical care.

In July 2018, we launched the inpatient oncology narrative medicine workshop. Facilitators reviewed Narrative Medicine theory, and participants read two thematically-relevant pieces (prose or poetry) regarding the clinical oncology experience of patients and providers. Residents then wrote a narrative for 10–20 min about an experience on their current rotation. We chose more prescriptive content and prompts than typical narrative medicine workshops to directly engage team members about their current experience. Residents were encouraged, but not required, to share their work aloud, and collectively discuss their experiences, motivations, feelings and viewpoints. If residents elected not to share, faculty facilitated discussions about the writing process or general resident experiences. Residents were encouraged to continue their pieces independently after the workshop with ongoing editing and support from facilitators.

### Program evaluation, residents

During Academic Year (AY) 2017–2018 (pre-implementation/ historical control) and AY2018–2019 (implementation year), we surveyed residents regarding their well-being on the initial and final weeks of the oncology rotation using the Physician Well-Being Index (PWBI), with anonymous linked identifiers. During implementation year, only 5 medical students participated joined the rotation and were not included in this analysis. The PWBI asks seven yes/no questions assessing multiple well-being domains (range 0–7, ≥4 corresponds to low well-being) [13] The Med-X rotation was structurally and educationally the same in both years. During control and implementation years, overall program burnout/well-being remained unchanged (verified informal communication).

### Program evaluation, faculty

Since we guaranteed participant anonymity, we conducted semi-structured interviews with workshop facilitators and the Med X program director for 30–60 min with a qualitative researcher (MS) and with two internal medicine program directors via email. Facilitators answered prompts regarding development/implementation challenges, differences between inpatient/outpatient workshops, and lessons learned. Facilitators were encouraged to share specifics of what they observed or what others had shared during the process of implementation. Program directors answered prompts regarding program response to burnout, including viewpoints about narrative medicine. The interviewer took notes using the respondent’s own words and used a grounded theory approach to identify relevant themes regarding inpatient narrative medicine/implementation. The interviewer then met with facilitators to re-explore themes, identify critical challenges and success factors, including facilitator competencies needed to successfully conduct narrative medicine workshops.

## Results

### Participants

**Fig. 1:**
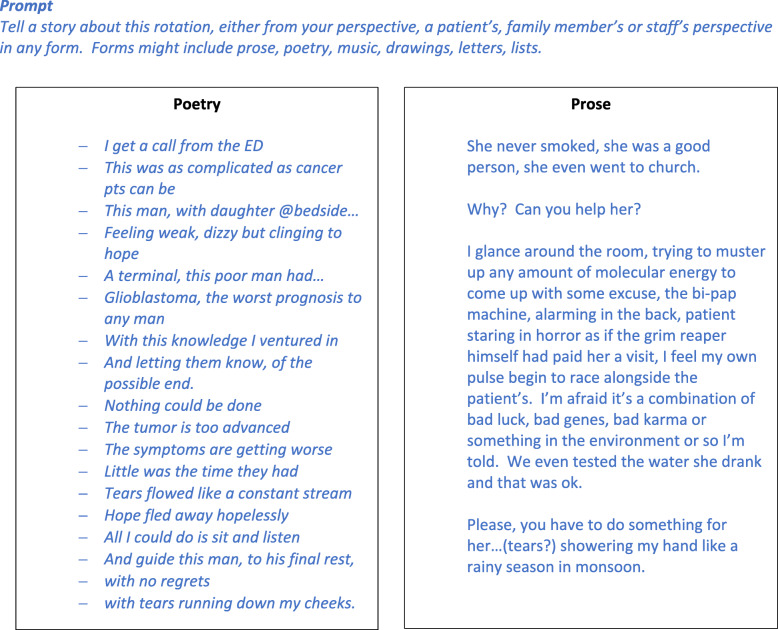
Examples of resident writing created during narrative oncology workshop *(shared with permission)*

Over AY2018–2019, 36 medical residents completed 12 monthly inpatient oncology narrative medicine workshops, including 16 senior residents and 20 junior residents/interns. In all but one workshop, participants voluntarily shared their written narratives during discussion. Typically, two or all three residents shared their narrative work. Residents wrote about viewpoints about mortality, patient experiences of illness and death, challenging situations, and points of conflict. Two pieces are shared with permission (Fig. 1).

#### Programmatic outcomes

##### Program implementation challenges and success factors

**Fig. 2:**
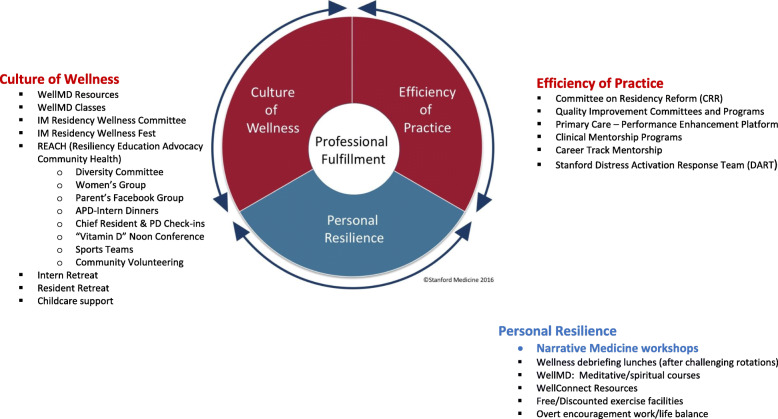
Narrative medicine workshops contextualized in a systematic approach to addressing Stanford medicine residents’ wellness.

Based on positive resident feedback, the residency program has continued to renew the oncology narrative medicine workshop series for an additional 3 years. In addition to the initial developmental principles, stakeholder interviews revealed additional themes relevant to program success:

- **Building trust:** *“I wasn’t sure it would work.” (program director).* Significance: Initially, program leadership was skeptical that that a narrative medicine workshop could be conducted during a clinically overwhelming rotation, as writing requires some mental room to think and reflect. However, the 7-year outpatient narrative medicine curriculum built sufficient trust with program leadership to allow a trial year.
- **Med-X floor staff partnership:** *“They became our champions.” (facilitator)* Protecting resident’s time was paramount to successful implementation, and critical patient care issues arise frequently during the oncology rotation. Significance: A unit secretary became an internal champion, developed a strategy to triage clinical requests, hold pages, and proactively have questions addressed before the workshop began.
- **Team leadership:** *“Sounds like a hard month” (senior resident statement, per facilitator).* Each month, a single team would come together for the workshop. Significance: Intern degree of participation and openness was largely dependent upon senior residents’ role modeling sharing behaviors.
- **Uncovering team dynamics:** *“You could tell what was happening in the team, based on what they shared.” (facilitator).* Significance: Teams were in various stages of burnout. Team dynamics, including support by senior resident and attending, were evident to the facilitators. Facilitators noted that these workshops could be “a canary in a cage” to help teams identify and mitigate burnout, including working with program leadership on systems changes to promote wellness.
- **Inpatient vs outpatient narrative medicine:** *“This was very different than outpatient workshops … the team aspect made a difference” (facilitator).* Significance: Outpatient narrative medicine workshops would bring together residents/medical students with different experiences on various rotations. The inpatient workshop was team-focused, allowing residents to share common experiences, thus deepening the nature of the discussion of personal narratives. Writing about and sharing a common experience allowed residents to additionally debrief in a way that is unique to an inpatient curriculum with an active team. This was not initially anticipated when the curriculum was created, but became a clear additional benefit to the residents.
- **Team release valve:** *“This gives team members an opportunity to reflect together, and share what was hard.” (facilitator).* Significance: Teams used the workshop to talk to each other personally and to process what they were experiencing – which they rarely could do during the daily press of work. Many specifically expressed gratitude for sharing their emotions/ experiences, and for ensuing peer-to-peer mentorship. Facilitators observed that some residents with “scientific mindsets,” previously skeptical of narrative medicine, seemed more receptive on their oncology rotation.
- **Value of trained facilitators:** *“You need to be ready for any emotion, and to guide participants”* (*facilitator*). More so than other rotations, inpatient oncology involves daily decisions about life, death, and sharing critical news. Significance: Facilitators discussed the need for training in moderating emotionally charged discussions, including creating a safe space for residents, holding space for strong emotions, and bringing the group to a thoughtful close after personal/emotional narratives were shared. When strong emotions or serious issues arose during the workshops, facilitators respected participant confidentiality, followed up with them personally, and provided access to residency resources for additional mental health support.
- **Important wellness tool, amongst others:** “*It’s time versus intensity. And, personal resiliency is important, but so is system support.*” (*program director*). Significance: Program directors discussed infrastructural challenges with emotionally taxing rotations, the critical role of the inpatient attending in minding the emotional health of their teams, shared other Stanford wellness resources, and considered how to implement other personal resiliency-oriented and systems-level interventions to support resident wellness while still meeting ACGME educational demands. Figure 2 is a conceptual framework of physician wellness from Stanford’s WellMD model, and we include this to acknowledge that our intervention is only one small part of many types of interventions required to support wellness.

#### Learner wellbeing/burnout outcomes

**Fig. 3:**
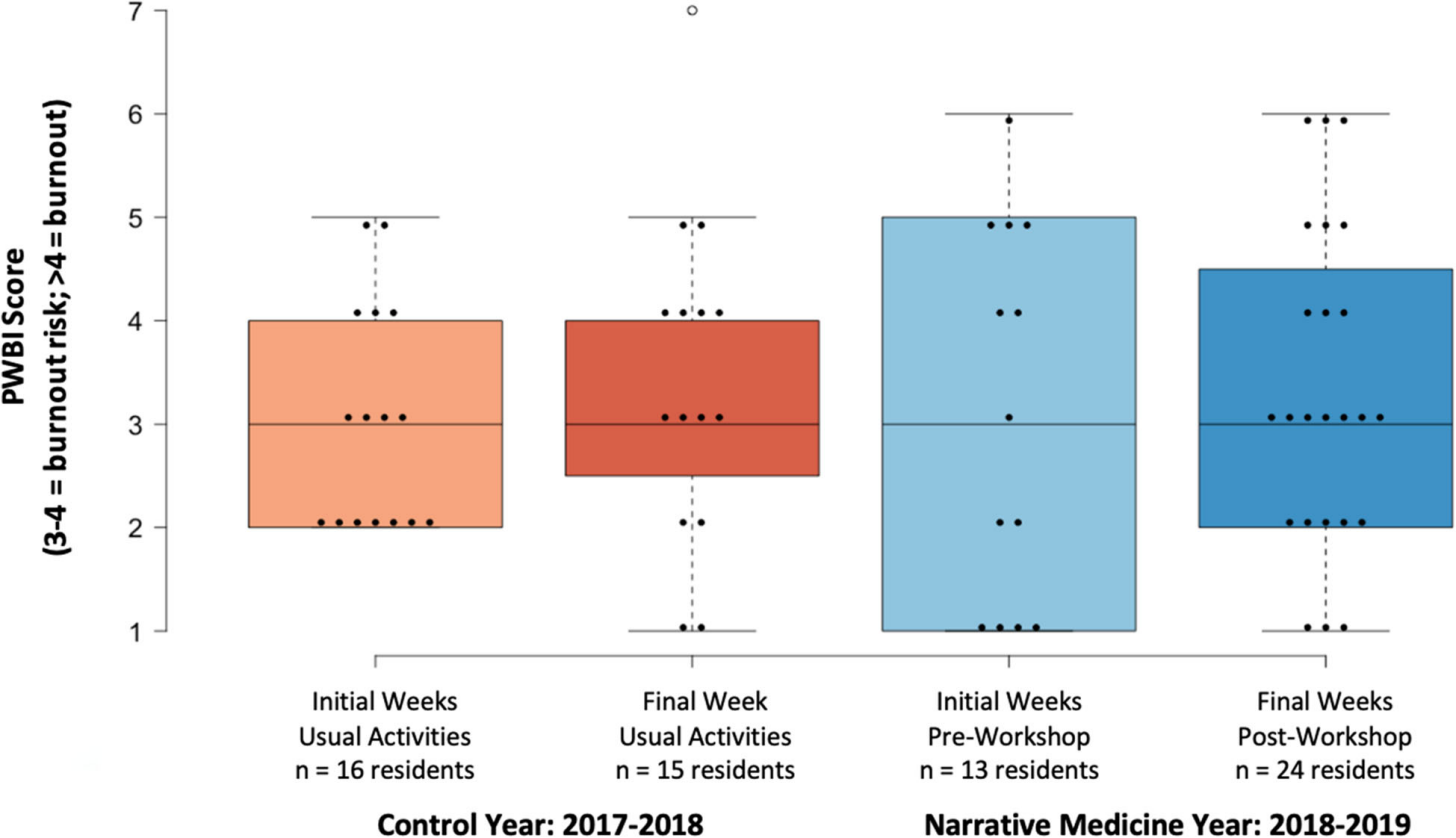
Short term impact of narrative medicine workshops on inpatient oncology: Effect of Narrative Medicine workshops on Physician Wellbeing Inventory (PWBI) scores of residents during an inpatient oncology rotation, in control (2017–2018) and workshop implementation (2018–2019) years

Overall, 30–70% of residents completed surveys each year (Fig. 3). During control year AY2017–2018, residents’ average wellbeing decreased by 13% from beginning (mean 3.0, SD 1.1, *n* = 16) to end (mean 3.4, SD 1.6, *n* = 15) of the rotation. During narrative medicine year AY2018–2019, resident wellbeing was decreased slightly less by 6% from initial weeks (mean 3.1, SD 1.9, *n* = 13) to final week (mean 3.3, SD 1.6, *n* = 24). Each year, 5 residents completed both pre- and post-surveys (Appendix A). Matched control year residents had decrement in PWBI (mean 2.4 to 3.8), while matched intervention residents had less decrement (mean 2.2 to 2.8). The program was underpowered to detect differences of less than 1.0 points on the 7-point PWBI scale (Appendix A).

#### Generalizable narrative medicine framework

##### Approach to session challenges

**Fig. 4:**
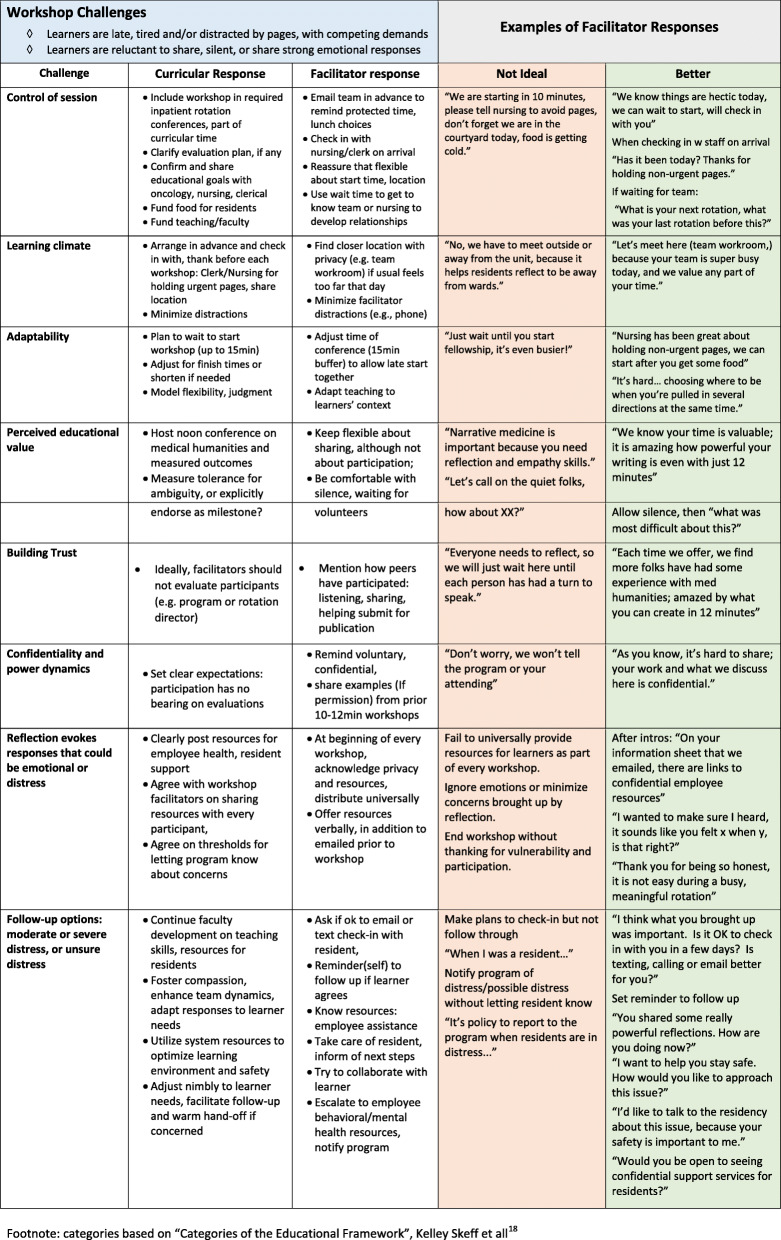
Program and facilitator responses to common workshop challenges. Footnote: categories based on “Categories of the Educational Framework”, Kelley Skeff et all [12]

After thematic review, facilitators identified approaches to common narrative medicine session challenges, including examples of non-ideal and better facilitator responses (Fig. 4). The taxonomy of Fig. 4 originates from Skeff’s “Categories of the Educational Framework” [12] and is expanded upon from facilitator experience with real world examples and the qualitative data. Additional narrative medicine teaching resources are shared in Supplemental Appendix B (typical inpatient session format), Appendix C (potential readings), and Appendix D (universal and focused prompts).

##### Narrative medicine facilitator competencies

**Fig. 5:**
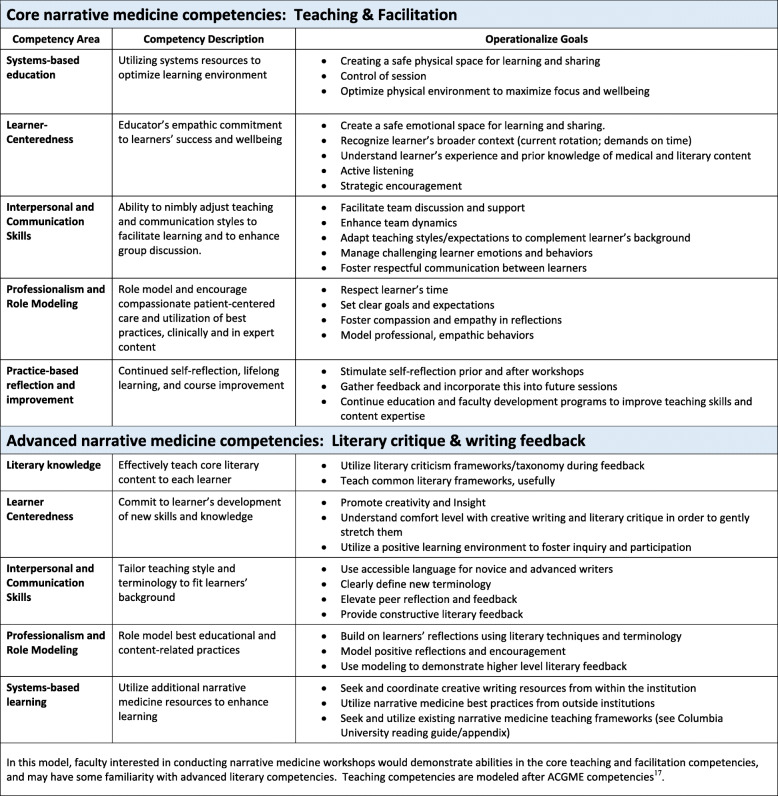
Core and advanced competencies for faculty facilitators of narrative medicine workshops

We identified core/essential competencies (facilitation and teaching) and advanced, non-core/non-essential competencies (literary critique and writing feedback) for facilitators to conduct narrative medicine workshops (Fig. 5). We expanded the “Teaching Competencies” framework [14] for narrative medicine facilitation based on literature review [15–17], our experiences, and analysis of the themes from the qualitative interviews. Core competencies were separated from advanced competencies based on group consensus.

## Discussion

We successfully implemented a narrative medicine workshop during an intensive inpatient oncology rotation for medical residents that may mitigate short-term burnout. Implementation success hinged on program support from residency leadership, nursing, unit secretaries, and trained facilitators. During workshops, residents had the opportunity to process their emotions and to come together as a team.

This educational intervention, while brief, adds to current understanding about feasibility of conducting reflective exercises during busy inpatient rotations by giving a clear roadmap and explanation of the elements of the program that made it successful. This program and study have several limitations. Burnout is a complex phenomenon with interplay between system factors and personal resilience [18]. From a well-being perspective, narrative medicine focuses primarily on improving personal resilience, and a single workshop is unlikely to have long term effects in reducing burnout, as any gains would likely attenuate quickly. The data we collected was underpowered; more studies are needed to more thoroughly assess our workshop’s effect on wellbeing. To meaningfully reduce burnout, institutions must enact system changes (such as protected time-off and appropriate workloads) and increased resiliency-focused activities to promote sustained wellness (Fig. 3) [4]. Second, while we identified core facilitation competencies that are familiar to most medical education faculty, some of the advanced literary competencies may require additional training (Fig. 5). Our goal is to present an accessible curriculum that in our experience was helpful to, and well-received by, busy residents. As such, the advanced literary competencies should not deter faculty from conducting similar workshops. When available, deeper literary critiques and perspectives will enrich the workshops by elevating the discussion of the texts and resident writings. Shared resources and collaborations with individuals/institutions with prior experiences can also help bridge these gaps. Third, while we used a meditative outdoor space to enhance learning climate, these workshops can occur in any private, comfortable conference room, or even via video. Finally, workshop facilitators were enthusiastic and positively biased about narrative medicine, potentially overestimating its impact. However, program leadership received enough positive direct feedback from residents to renew the workshop in the formal inpatient oncology curriculum for the last 3 years.

Even during the busiest and most emotionally challenging rotations, taking time to reflect and process can be a valuable experience [6, 19, 20]. Narrative medicine has the potential to promote well-being in medical professionals, by connecting clinicians to their values and ideals [3, 6]. The team-based nature of inpatient narrative medicine has further potential to improve cohesion and peer support [21, 22]. Moving forward, we hope to expand the narrative medicine program into other critical inpatient rotations, including multi-disciplinary groups, where teams need an outlet to process, and unveil new truths about their experiences.

## Supplementary Information

**Additional file 1.**

**Additional file 2.**

**Additional file 3.**

**Additional file 4.**

## Data Availability

All data generated or analyzed during this study are included in this published article [and its supplementary information files].
