# Supplementary material for: When it’s needed most: a blueprint for resident creative writing workshops during inpatient rotations
Source: BMC Med Educ. 2021 Oct 20;21:535. doi: 10.1186/s12909-021-02935-x (PMC8529814; doi:10.1186/s12909-021-02935-x)
Supplement: Supplementary file 1 — Additional file 1. [file 12909_2021_2935_MOESM1_ESM.docx]

**When it’s Needed Most: A Blueprint for Resident Creative Writing Workshops during Inpatient Rotations**

Lauren Michelle Edwards, MD^1^; Yeuen Kim, MD^1^; Matthew Stevenson, MD^2^; Tyler Johnson, MD^3^; Nora Sharp^4,5^; Anna Reisman, MD6; Malathi Srinivasan, MD^1,4^

1. Division of Primary Care and Population Health, Stanford School of Medicine, Palo Alto, CA
2. Division of Primary Care, Palo Alto Veterans Administration Hospital, Palo Alto, CA
3. Division of Hematology and Oncology, Stanford School of Medicine, Palo Alto, CA
4. Stanford Center for Asian Health Research and Education, Stanford School of Medicine, Palo Alto, CA
5. Computational and Systems Biology Interdepartmental Program, University of California, Los Angeles, Westwood, CA
6. Department of Internal Medicine (General Medicine), Yale School of Medicine, New Haven, CT

**Corresponding Author Lauren Michelle Edwards, MD**

Program Co-Director, Narrative Medicine

Assistant Clinical Professor

Division of Primary Care and Population Health

Stanford University School of Medicine

960 North San Antonio Road, Suite 101

Los Altos, CA 94022

Work: 650-498-9000

Mobile: 510-295-9891

laurened@stanford.edu

**Author Affiliations and Contributions**

**Lauren Michelle Edwards, MD**

Program Co-Director, Narrative Medicine

Assistant Clinical Professor

Division of Primary Care and Population Health

Stanford University School of Medicine

***Contributions:*** program design and implementation, study design, manuscript preparation

**Yeuen Kim, MD MAS**
Program Co-Director, Narrative Medicine

Clinical Instructor

Division of Primary Care and Population Health

San Francisco Department of Public Health, Outbreak Management Group

***Contributions:*** program design and implementation, study design, manuscript preparation

**Matthew Stevenson, MD**

Program Co-Director, Narrative Medicine

Clinical Assistant Professor

Division of Primary Care and Population Health

Palo Alto Veterans Administration Hospital

***Contributions:*** program design and implementation, study design, manuscript preparation

**Tyler Johnson, MD**

Program Director, Oncology Residency Training Program

Associate Clinical Professor

Division of Hematology and Oncology

Stanford University School of Medicine

***Contributions:*** program design and implementation, manuscript preparation

**Nora Sharp**

Program Administrator

Center for Asian Health Research and Education

Stanford University School of Medicine

Computational and Systems Biology Interdepartmental Program, University of California, Los Angeles

***Contributions:*** study design, data analysis and interpretation, manuscript preparation

**External Expert**

**Anna Reisman, MD**

Professor of Medicine, Yale University School of Medicine

Director, Yale School of Medicine Program for Humanities in Medicine

***Contributions:*** data analysis and interpretation, manuscript preparation

**Malathi Srinivasan, MD**

Clinical Professor of Medicine

Division of Primary Care and Population Health

Stanford University School of Medicine

***Contributions:*** Study design, qualitative data analysis, manuscript preparation

Online Appendix A

1. Effect of Narrative Medicine workshops on Physician Wellbeing Inventory (PWBI) and PWBI items scores of residents during an inpatient oncology rotation, in control (2017-2018) and workshop implementation (2018-2019) years

|  | **2017-2018 Academic Year**  **Without Narrative Medicine Curriculum** | | | **2018-2019 Academic Year**  **With Narrative Medicine Curriculum** | | |  |
| --- | --- | --- | --- | --- | --- | --- | --- |
|  | **Mean Initial Weeks (SD)**  **N=16** | **Mean Final Week (SD)**  **N=15** | **Mean Change Between Initial and Final Weeks**  **(P-Value of Initial Weeks vs Final Week)** | **Mean Initial Weeks (SD)**  **N=13 ^b^** | **Mean Final Week (SD)**  **N=24** | **Mean Change Between Initial and Final Weeks**  **(P-Value of Initial Weeks vs Final Week)** | **P-Value of Mean Difference Between Control and Implementation Years** |
| Total PWBI | 3.0 (1.1) | 3.40 (1.60) | 0.40 (0.42) | 3.08 (1.85) | 3.29 (1.57) | 0.21 (0.71) | 0.62 |
| PWBI – Burnout | 0.81 (0.40) | 1 (0) | 0.19 (0.08) | 0.62 (0.51) | 0.83 (0.38) | 0.21 (0.15) | 0.74 |
| PWBI – Emotional Detachment | 0.75 (0.45) | .60 (0.51) | -0.15 (0.39) | 0.62 (0.51) | 0.75 (0.44) | 0.13 (0.41) | 0.02 |
| PWBI – Depression | 0.19 (0.40) | .33 (0.49) | 0.14 (0.37) | 0.54 (0.52) | 0.42 (0.50) | -0.12 (0.49) | 0.03 |
| PWBI – Fatigue | 0.06 (0.25) | 0.07 (0.26) | 0.01 (0.96) | 0.15 (0.38) | 0.17 (0.38) | 0.02 (0.92) | 0.91 |
| PWBI – Stress/Overload | 0.38 (0.50) | 0.40 (0.51) | 0.02 (0.89) | 0.38 (0.51) | 0.25 (0.44) | -0.13 (0.41) | 0.18 |
| PWBI – Anxiety & Irritability | 0.69 (0.48) | 0.80 (0.41) | 0.11 (0.49) | 0.54 (0.52) | 0.58 (0.50) | 0.04 (0.80) | 0.57 |
| PWBI – Physical Quality of Life  Decrease | 0.13 (0.34) | 0.20 (0.41) | 0.07 (0.59) | 0.25 ^c^ (0.45) | 0.29 (0.46) | 0.04 (0.80) | 0.75 |
| 1. Residents took the PWBI survey at the beginning and end of the rotations. PWBI of 3-4 indicates a high risk for burnout, whereas PWBI of 4 or greater indicates burnout. 2. One individual pre rotation, 2018-2019, did not answer for PWBI - Physical Quality of Life, and that mean is calculated out of 12 individuals 3. The first survey for each person was coded as “Initial Weeks” survey if done. One individual in 2018-2019 completed their initial survey in Week 3 and second survey post-workshop in Week 3. Another individual responded in 2018-2019 completed their initial survey in Week 3 and second survey post-workshop in Week 4. 4. Table includes information from all surveys from 2017-2018 and 2018-2019 with complete PWBI information for initial and final survey by medicine residents on Stanford inpatient oncology rotation. | | | | | | | |

1. Effect of Narrative Medicine Curriculum on Physician Wellbeing Inventory (PWBI) scores of residents during an inpatient oncology rotation, in initial and final week paired samples for control (2017-2018) and workshop implementation (2018-2019) Years

|  | **2017-2018 Academic Year**  **Without Narrative Medicine Curriculum** | | | **2018-2019 Academic Year**  **With Narrative Medicine Curriculum** | | |  |
| --- | --- | --- | --- | --- | --- | --- | --- |
| **Measure** | **Mean Initial Week (SD)**  **N=5** | **Mean Final Week (SD)**  **N=5** | **Mean Change Between Initial and Final Weeks**  **(P-Value of Initial Weeks vs Final Week)** | **Mean Initial Week (SD)**  **N=5** | **Mean Final Week (SD)**  **N=5** | **Mean Change Between Initial and Final Weeks**  **(P-Value of Initial Weeks vs Final Week)** | **P-Value of Mean Difference Between Control and Implementation Years** |
| Total PWBI | 2.4 (0.89) | 3.8 (0.84) | 1.4 (0.005) | 2.2 (2.17) | 2.8 (1.30) | 0.60 (0.21) | 0.22 |
| PWBI – Burnout | 0.8 (0.45) | 1 (0) | 0.2 (0.37) | 0.60 (0.55) | 0.80 (0.45) | 0.20 (0.37) | 1 |
| PWBI – Emotional Detachment | 1 (0) | 1 (0) | 0 (N/A) | 0.40 (0.55) | 0.40 (0.55) | 0 (N/A) | 1 |
| PWBI – Depression | 0 (0) | 0.2 (0.45) | 0.2 (0.37) | 0.20 (0.45) | 0.40 (0.55) | 0.20 (0.37) | 1 |
| PWBI – Fatigue | 0 (0) | 0 (0) | 0 (N/A) | 0.20 (0.45) | 0 (0) | -0.20 (0.37) | 0.06 |
| PWBI – Stress/Overload | 0.2 (0.45) | 0.6 (0.55) | 0.4 (0.18) | 0.40 (0.55) | 0.40 (0.55) | 0 (1) | 0.11 |
| PWBI-Anxiety and Irritability | 0.4 (0.55) | 1 (0) | 0.6 (0.07) | 0.40 (0.55) | 0.60 (0.55) | 0.20 (0.37) | 0.08 |
| PWBI-Physical Quality of Life Decrease | 0 (0) | 0 (0) | 0 (N/A) | 0 (0) | 0.20 (0.45) | 0.20 (0.37) | 0.06 |
| 1. Table includes information from surveys from 2017-2018 and 2018-2019 with complete PWBI information for paired initial and final surveys by medicine residents on Stanford inpatient oncology rotation. | | | | | | | |

3. Graphic: Effect of Narrative Medicine Curriculum on Physician Wellbeing Inventory (PWBI) scores of residents during an inpatient oncology rotation, in initial and final week paired samples for control (2017-2018) and workshop implementation (2018-2019) Years


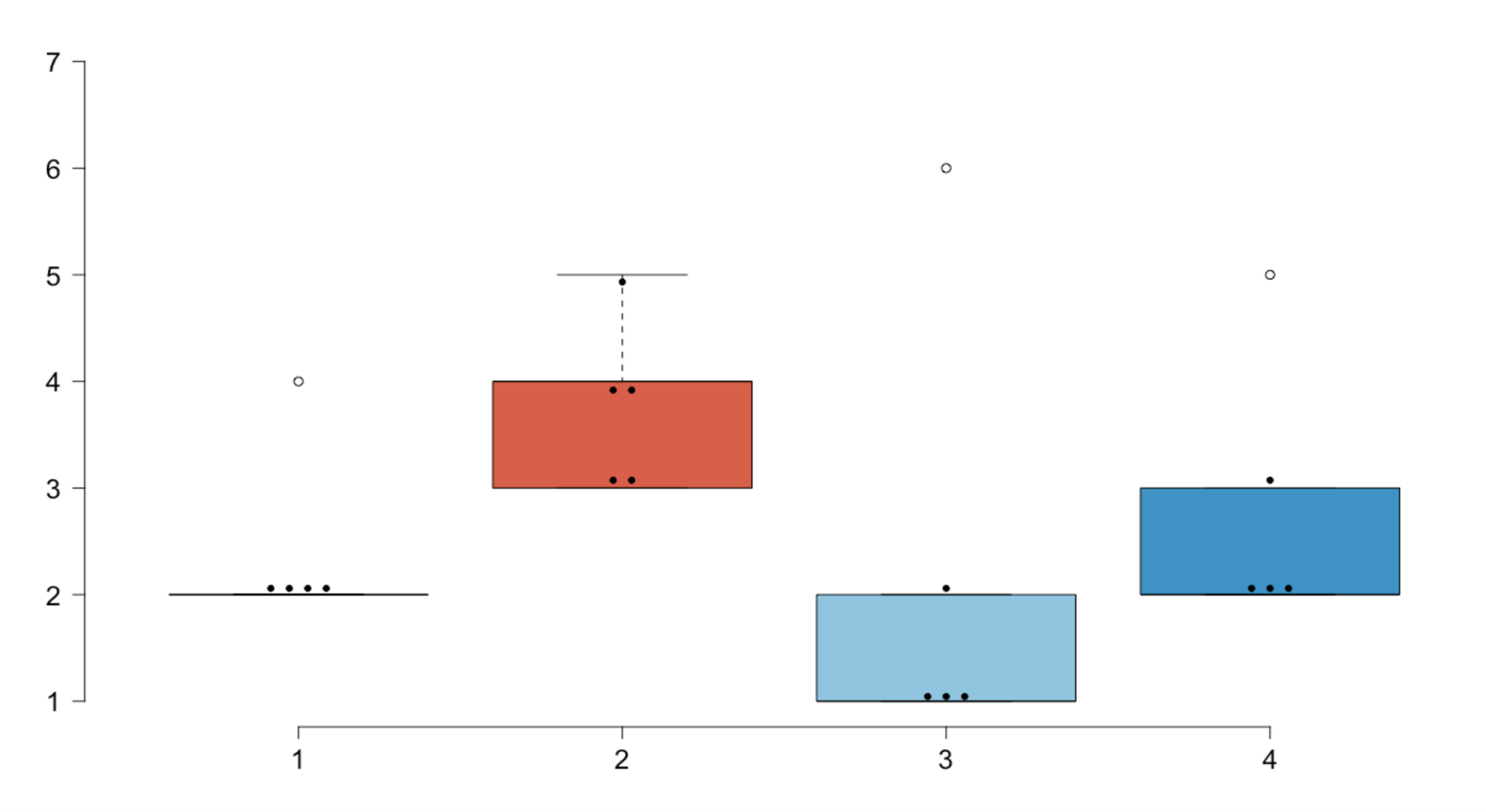


**Narrative Medicine Year: 2018-2019**

**Control Year: 2017-2018**

Final Weeks Post-Workshop n = 5 residents

Initial Weeks Pre-Workshop n = 5 residents

Initial Weeks Usual Activities n = 5 residents

Final Week Usual Activities n = 5 residents

**PWBI SCORE**

**(3-4 = burnout risk; >4 = burnout)**
