# Supplementary material for: When it’s needed most: a blueprint for resident creative writing workshops during inpatient rotations
Source: BMC Med Educ. 2021 Oct 20;21:535. doi: 10.1186/s12909-021-02935-x (PMC8529814; doi:10.1186/s12909-021-02935-x)
Supplement: Supplementary file 2 — Additional file 2. [file 12909_2021_2935_MOESM2_ESM.docx]

**When it’s Needed Most: A Blueprint for Resident Creative Writing Workshops during Inpatient Rotations**

Lauren Michelle Edwards, MD^1^; Yeuen Kim, MD^1^; Matthew Stevenson, MD^2^; Tyler Johnson, MD^3^; Nora Sharp^4,5^; Anna Reisman, MD6; Malathi Srinivasan, MD^1,4^

1. Division of Primary Care and Population Health, Stanford School of Medicine, Palo Alto, CA
2. Division of Primary Care, Palo Alto Veterans Administration Hospital, Palo Alto, CA
3. Division of Hematology and Oncology, Stanford School of Medicine, Palo Alto, CA
4. Stanford Center for Asian Health Research and Education, Stanford School of Medicine, Palo Alto, CA
5. Computational and Systems Biology Interdepartmental Program, University of California, Los Angeles, Westwood, CA
6. Department of Internal Medicine (General Medicine), Yale School of Medicine, New Haven, CT

**Corresponding Author Lauren Michelle Edwards, MD**

Program Co-Director, Narrative Medicine

Assistant Clinical Professor

Division of Primary Care and Population Health

Stanford University School of Medicine

960 North San Antonio Road, Suite 101

Los Altos, CA 94022

Work: 650-498-9000

Mobile: 510-295-9891

laurened@stanford.edu

**Author Affiliations and Contributions**

**Lauren Michelle Edwards, MD**

Program Co-Director, Narrative Medicine

Assistant Clinical Professor

Division of Primary Care and Population Health

Stanford University School of Medicine

***Contributions:*** program design and implementation, study design, manuscript preparation

**Yeuen Kim, MD MAS**
Program Co-Director, Narrative Medicine

Clinical Instructor

Division of Primary Care and Population Health

San Francisco Department of Public Health, Outbreak Management Group

***Contributions:*** program design and implementation, study design, manuscript preparation

**Matthew Stevenson, MD**

Program Co-Director, Narrative Medicine

Clinical Assistant Professor

Division of Primary Care and Population Health

Palo Alto Veterans Administration Hospital

***Contributions:*** program design and implementation, study design, manuscript preparation

**Tyler Johnson, MD**

Program Director, Oncology Residency Training Program

Associate Clinical Professor

Division of Hematology and Oncology

Stanford University School of Medicine

***Contributions:*** program design and implementation, manuscript preparation

**Nora Sharp**

Program Administrator

Center for Asian Health Research and Education

Stanford University School of Medicine

Computational and Systems Biology Interdepartmental Program, University of California, Los Angeles

***Contributions:*** study design, data analysis and interpretation, manuscript preparation

**External Expert**

**Anna Reisman, MD**

Professor of Medicine, Yale University School of Medicine

Director, Yale School of Medicine Program for Humanities in Medicine

***Contributions:*** data analysis and interpretation, manuscript preparation

**Malathi Srinivasan, MD**

Clinical Professor of Medicine

Division of Primary Care and Population Health

Stanford University School of Medicine

***Contributions:*** Study design, qualitative data analysis, manuscript preparation

**Appendix B**

**Suggested format of a 45-minute Narrative Medicine Inpatient workshop**

| **Time*** | **Purpose** | **Description** |
| --- | --- | --- |
| 00:00 | Establish learning climate | Welcome, establish ground rules to create a safe space, distribute lunches, brief introductions |
| 00:05 | Priming activity | Ask participants to volunteer to read 2 pieces to prompt reflection; take turns reading aloud by stanza or a few paragraphs at a time |
| 00:15 | Promote reflection | Ask for a 2-3 sentence synopsis of prose/poem, observations e.g. images they noticed, phrases that stood out, any reminders of own experiences, what they liked least/most |
| 00:20 | Reflective writing | Set timer for 10-12min, offer prompt, remind that any form of story e.g. lists, phrases, quotes are some ways prior residents have written about their rotation; at mid-point give 5min reminder. Facilitator should also write because it models and replicates difficulty |
| 00:30 | Share reflections | Ask if anyone found writing difficult, if any part was a struggle; ask for volunteers to read their writing. Allow silence, wait for volunteers. If some starts talking about the writing, ask them to read directly from what they wrote, thanking them for vulnerability and sharing their work. |
| 00:40 | Facilitated debriefing | Similar to observations/reflections on readings, ask: what did you notice, why do you say that, what else, did that sound familiar? |
| 00:45 | Closing and follow-up | Thank everyone for participating and their time, offer resources again for wellness. If difficult topic or conversation, ask participants who had difficulty before they leave if ok to follow up with them, e.g. email/text/phone. If concerned, explain to participant that you are concerned for their safety, that you are going to ask for help making sure the participant is safe. Follow up as appropriate with participant, program. |

*We use flexible start times in case the team is running late, so while the workshop runtimes are rough guidelines; we suggest allowing an extra 15min before starting to accommodate time spent gathering team, finishing family meetings, etc.
