# Supplementary material for: When it’s needed most: a blueprint for resident creative writing workshops during inpatient rotations
Source: BMC Med Educ. 2021 Oct 20;21:535. doi: 10.1186/s12909-021-02935-x (PMC8529814; doi:10.1186/s12909-021-02935-x)
Supplement: Supplementary file 3 — Additional file 3. [file 12909_2021_2935_MOESM3_ESM.docx]

**When it’s Needed Most: A Blueprint for Resident Creative Writing Workshops during Inpatient Rotations**

Lauren Michelle Edwards, MD^1^; Yeuen Kim, MD^1^; Matthew Stevenson, MD^2^; Tyler Johnson, MD^3^; Nora Sharp^4,5^; Anna Reisman, MD6; Malathi Srinivasan, MD^1,4^

1. Division of Primary Care and Population Health, Stanford School of Medicine, Palo Alto, CA
2. Division of Primary Care, Palo Alto Veterans Administration Hospital, Palo Alto, CA
3. Division of Hematology and Oncology, Stanford School of Medicine, Palo Alto, CA
4. Stanford Center for Asian Health Research and Education, Stanford School of Medicine, Palo Alto, CA
5. Computational and Systems Biology Interdepartmental Program, University of California, Los Angeles, Westwood, CA
6. Department of Internal Medicine (General Medicine), Yale School of Medicine, New Haven, CT

**Corresponding Author Lauren Michelle Edwards, MD**

Program Co-Director, Narrative Medicine

Assistant Clinical Professor

Division of Primary Care and Population Health

Stanford University School of Medicine

960 North San Antonio Road, Suite 101

Los Altos, CA 94022

Work: 650-498-9000

Mobile: 510-295-9891

laurened@stanford.edu

**Author Affiliations and Contributions**

**Lauren Michelle Edwards, MD**

Program Co-Director, Narrative Medicine

Assistant Clinical Professor

Division of Primary Care and Population Health

Stanford University School of Medicine

***Contributions:*** program design and implementation, study design, manuscript preparation

**Yeuen Kim, MD MAS**
Program Co-Director, Narrative Medicine

Clinical Instructor

Division of Primary Care and Population Health

San Francisco Department of Public Health, Outbreak Management Group

***Contributions:*** program design and implementation, study design, manuscript preparation

**Matthew Stevenson, MD**

Program Co-Director, Narrative Medicine

Clinical Assistant Professor

Division of Primary Care and Population Health

Palo Alto Veterans Administration Hospital

***Contributions:*** program design and implementation, study design, manuscript preparation

**Tyler Johnson, MD**

Program Director, Oncology Residency Training Program

Associate Clinical Professor

Division of Hematology and Oncology

Stanford University School of Medicine

***Contributions:*** program design and implementation, manuscript preparation

**Nora Sharp**

Program Administrator

Center for Asian Health Research and Education

Stanford University School of Medicine

Computational and Systems Biology Interdepartmental Program, University of California, Los Angeles

***Contributions:*** study design, data analysis and interpretation, manuscript preparation

**External Expert**

**Anna Reisman, MD**

Professor of Medicine, Yale University School of Medicine

Director, Yale School of Medicine Program for Humanities in Medicine

***Contributions:*** data analysis and interpretation, manuscript preparation

**Malathi Srinivasan, MD**

Clinical Professor of Medicine

Division of Primary Care and Population Health

Stanford University School of Medicine

***Contributions:*** Study design, qualitative data analysis, manuscript preparation

**Appendix C**

**Inpatient Narrative Medicine Workshops: Examples of Short Prose and Poetry Readings to Stimulate Discussion**

We used medically themed readings to stimulate discussion. However, any insightful literary reading on a theme encountered in clinical medicine would be appropriate.

| **Theme** | **Author** | **Title** | **Description** |
| --- | --- | --- | --- |
| **Hope** | Andrea M. Watson | “Hope Shattered, Hope Restored” | A narrative on the role and power of imperfect hope and survival of an oncology patient |
| **Humor** | Donald Hall | “Ship Pounding” | Poem, brief humor comparing healthy and sick |
| **Humor** | William Meffert | “Incoming” | Short story, surgeon puts own life at risk to remove grenade from young soldier |
| **“Difficult” Patients** | Michael Lewis | “Home Game” (excerpt) | Narrative, in which an absentee father navigates the dehumanizing aspects of a child’s bronchiolitis hospitalization |
| **Setting** | Laurie Kutchins | “The Ward is the World” | Narrative, reflecting on the disruptions to time and space that occur when undergoing cycles of chemotherapy |
| **Grief** | Minjee Kim | “The Right to Grieve” | Narrative, discussing the rights of physicians to feel a full range of emotions after patient death |
| **Grief** | Jennifer Lycette | “Making Room” | Narrative, how to find space as a provider for your own feelings around losing a patient |
| **Illness** | Nikta Raman | “DNA” | Poem, describing the feeling of betrayal of one’s own DNA when cancer develops |
| **Loss** | Jane Kenyon | "The Sick Wife” | Poem, describing own illness from outsider’s point of view, sitting in car at odd hours of the day (like interns on day off) |
|  | Elizabeth Bishop | “One Art” | Poem, art of losing: self, relationships |
| **Perception, conflicting viewpoints** | Donald Hall | “Ship Pounding” | Poem, realizing hospital and docked ship are constantly working but going nowhere |
| **Boundaries, identity** | Rafael Campo | “The Abdominal Exam” | Poem, examination diagnosing terminal illness, powerless to change outcome |
| **Community** | William Meffert | “Incoming” | Short story, surgical team in field putting patient’s emergency ahead of own welfare |
| **Professional Identity** | Raymond Deng | “Performing Grief” | Narrative of medical student’s first code blue, with reflection on the “performance that permeates the profession” |
