# Supplementary material for: When it’s needed most: a blueprint for resident creative writing workshops during inpatient rotations
Source: BMC Med Educ. 2021 Oct 20;21:535. doi: 10.1186/s12909-021-02935-x (PMC8529814; doi:10.1186/s12909-021-02935-x)
Supplement: Supplementary file 4 — Additional file 4. [file 12909_2021_2935_MOESM4_ESM.docx]

**When it’s Needed Most: A Blueprint for Resident Creative Writing Workshops during Inpatient Rotations**

Lauren Michelle Edwards, MD^1^; Yeuen Kim, MD^1^; Matthew Stevenson, MD^2^; Tyler Johnson, MD^3^; Nora Sharp^4,5^; Anna Reisman, MD6; Malathi Srinivasan, MD^1,4^

1. Division of Primary Care and Population Health, Stanford School of Medicine, Palo Alto, CA
2. Division of Primary Care, Palo Alto Veterans Administration Hospital, Palo Alto, CA
3. Division of Hematology and Oncology, Stanford School of Medicine, Palo Alto, CA
4. Stanford Center for Asian Health Research and Education, Stanford School of Medicine, Palo Alto, CA
5. Computational and Systems Biology Interdepartmental Program, University of California, Los Angeles, Westwood, CA
6. Department of Internal Medicine (General Medicine), Yale School of Medicine, New Haven, CT

**Corresponding Author Lauren Michelle Edwards, MD**

Program Co-Director, Narrative Medicine

Assistant Clinical Professor

Division of Primary Care and Population Health

Stanford University School of Medicine

960 North San Antonio Road, Suite 101

Los Altos, CA 94022

Work: 650-498-9000

Mobile: 510-295-9891

laurened@stanford.edu

**Author Affiliations and Contributions**

**Lauren Michelle Edwards, MD**

Program Co-Director, Narrative Medicine

Assistant Clinical Professor

Division of Primary Care and Population Health

Stanford University School of Medicine

***Contributions:*** program design and implementation, study design, manuscript preparation

**Yeuen Kim, MD MAS**
Program Co-Director, Narrative Medicine

Clinical Instructor

Division of Primary Care and Population Health

San Francisco Department of Public Health, Outbreak Management Group

***Contributions:*** program design and implementation, study design, manuscript preparation

**Matthew Stevenson, MD**

Program Co-Director, Narrative Medicine

Clinical Assistant Professor

Division of Primary Care and Population Health

Palo Alto Veterans Administration Hospital

***Contributions:*** program design and implementation, study design, manuscript preparation

**Tyler Johnson, MD**

Program Director, Oncology Residency Training Program

Associate Clinical Professor

Division of Hematology and Oncology

Stanford University School of Medicine

***Contributions:*** program design and implementation, manuscript preparation

**Nora Sharp**

Program Administrator

Center for Asian Health Research and Education

Stanford University School of Medicine

Computational and Systems Biology Interdepartmental Program, University of California, Los Angeles

***Contributions:*** study design, data analysis and interpretation, manuscript preparation

**External Expert**

**Anna Reisman, MD**

Professor of Medicine, Yale University School of Medicine

Director, Yale School of Medicine Program for Humanities in Medicine

***Contributions:*** data analysis and interpretation, manuscript preparation

**Malathi Srinivasan, MD**

Clinical Professor of Medicine

Division of Primary Care and Population Health

Stanford University School of Medicine

***Contributions:*** Study design, qualitative data analysis, manuscript preparation

**Appendix D**

**Inpatient Narrative Medicine Workshops: Examples of Literary Prompts for Medical Learners**

**For our inpatient narrative medicine workshops, we used a single prompt (as shown in Figure 1), however, educators may choose from a variety of other writing prompts. In this table, we provide some examples of prompts to consider.**

| **Theme** | **Prompt/Questions to simulate reflection** |
| --- | --- |
| Universal storytelling | Tell a story about this rotation, either from your perspective, a patient’s, family member’s or staff’s perspective in any form. |
| Open-ended | -What story have you been wanting to tell?  -Write a letter to a patient or family member. |
| Boundaries, identity | The culture of medicine creates a professional identity for each of us as practitioners in the field. For many physicians, that culture may be distinct from the social identities formed long before professional training. Write a story about the intersection of your personal and professional identities in medicine. |
| Loss | Tell a story about the role of denial/disbelief/hope with a difficult prognosis. |
| Perception, conflicting viewpoints | The causes of illness can be understood in divergent and even contradictory ways, often leading to baffling and damaging differences between doctors and patients. Write a story about a time when you and a patient disagreed about the cause of his or her illness. |
| Joy | Tell a story of joy, in any form |
